# Post-laparoscopic cholecystectomy Mirizzi syndrome induced by polymeric surgical clips: a case report and review of the literature

**DOI:** 10.1186/s13256-016-0932-5

**Published:** 2016-05-27

**Authors:** Eleni-Aikaterini Nagorni, Georgios Kouklakis, Alexandra Tsaroucha, Soultana Foutzitzi, Nikos Courcoutsakis, Konstantinos Romanidis, Konstantinos Vafiadis, Michael Pitiakoudis

**Affiliations:** 1 Second Department of Surgery, Democritus University of Thrace, University Hospital of Alexandroupolis, Dragana, 68100 Alexandroupolis, Greece; 2 Gastrointestinal Endoscopy Unit, Democritus University of Thrace, University General Hospital of Alexandroupolis, Dragana, 68100 Alexandroupolis, Greece; 3 Department of Radiology and Medical Imaging, University Hospital of Alexandroupolis, Dragana, 68100 Alexandroupolis, Greece; 4 Department of Radiology, Didimotichon General Hospital, Didimotichon, 68300 Greece

**Keywords:** Clip migration, Endoscopic retrograde cholangiopancreatography, Laparoscopic cholecystectomy, Mirizzi syndrome, Polymer laparoscopic clip, Post-cholecystectomy syndrome

## Abstract

**Background:**

Laparoscopic cholecystectomy is the gold standard treatment of gallbladder disease. Post-cholecystectomy syndrome is a severe postoperative complication which can be caused by multiple mechanisms and can present with multiple disorders. The wide use of laparoscopy induces the need to understand more clearly the presentation and pathophysiology of this syndrome. Post-cholecystectomy Mirizzi syndrome is one form of this syndrome and, according to literature, this is the first report that clearly describes it.

**Case presentation:**

We describe the case of a 62-year-old Greek woman who underwent laparoscopic cholecystectomy because of gallstone disease. A few days after surgery, post-cholecystectomy syndrome gradually developed with mild bilirubin increase in association with epigastric pain, nausea, and vomiting. After performing ultrasound, magnetic resonance cholangiopancreatography, and endoscopic retrograde cholangiopancreatography, we conducted a second laparoscopic surgery to manage the obstruction, which was converted to open surgery because of the remaining inflammation from the post-endoscopic retrograde cholangiopancreatography acute pancreatitis. Four polymeric laparoscopic clips were removed because they were identified as the cause of her post-cholecystectomy syndrome. She had a quick recovery without further complications.

**Conclusions:**

Postoperative Mirizzi syndrome induced by the migration of polymer laparoscopic clips is a rare (only one case referring to polymeric clips has been published in the literature) but a well-identified complication of laparoscopic cholecystectomy which can confuse the diagnostic and therapeutic field requiring simultaneous immediate management.

## Background

Gallstone disease is one of the most common digestive diseases; it affects over 10 % of the general population and leads to many cholecystectomies annually. Approximately 20 to 40 % of patients with asymptomatic cholelithiasis will develop symptoms during their lifetime [1]. Laparoscopic cholecystectomy is the gold standard surgical treatment for cholelithiasis and is performed on 90 % of the patients with gallbladder disease in the USA. Despite its effectiveness, some postoperative complications are possible [2].

We use the term post-cholecystectomy syndrome (PCS) to describe a wide range of symptoms that present after cholecystectomy. Study-to-study variability on the incidence of PCS ranges from 10 to 40 %. Female patients have a 43 % risk for presenting with this syndrome whereas males have only a 28 % risk. The etiology of PCS is multiple and contains many pathophysiologies; therefore, PCS is a preliminary diagnosis which should be renamed with respect to the disease identified by a special investigation. PCS is characterized as early when it appears in the first postoperative days and as late when it appears months or years after laparoscopic cholecystectomy. Early PCS can be caused by biliary injury, retained cystic duct, or common bile duct stones inducing a postoperative Mirizzi syndrome. Late PCS can be due to recurrent common bile duct stones, bile duct strictures, or biliary dyskinesia of sphincter of Oddi [2].

We present a case of a 62-year-old woman who underwent laparoscopic cholecystectomy that was complicated by early post-cholecystectomy Mirizzi syndrome because of a polymeric laparoscopic clip, which immigrated during the first postoperative days causing obstruction of her common hepatic duct.

## Case presentation

A 62-year-old Greek woman presented to our hospital complaining about nonspecific, periodic, stinging pain to her right upper abdomen. She had already obtained an ultrasound (US) diagnosis of multiple gallbladder stones. Her medical history showed arterial hypertension and dyslipidemia but her surgical history was unremarkable. Her family history revealed her mother's chronic chololithiasis. During a clinical examination, tenderness in her right upper abdomen was revealed. Blood examinations were in normal range except for a mild increase in her lactate dehydrogenase (LDH) level (254 U/L; normal range 25 to 248 U/L).

After we obtained her consent, a standard laparoscopic cholecystectomy was performed. Her gallbladder was easily separated from her liver bed by using ligature systems and graspers. The laparoscopic clips that we used were of the polymeric type. We removed her bladder into an endobag through the umbilical port-site. The operation was completed without any perioperative complications. There was no leakage or bleeding so drainage did not have to be placed.

**Fig. 1:**
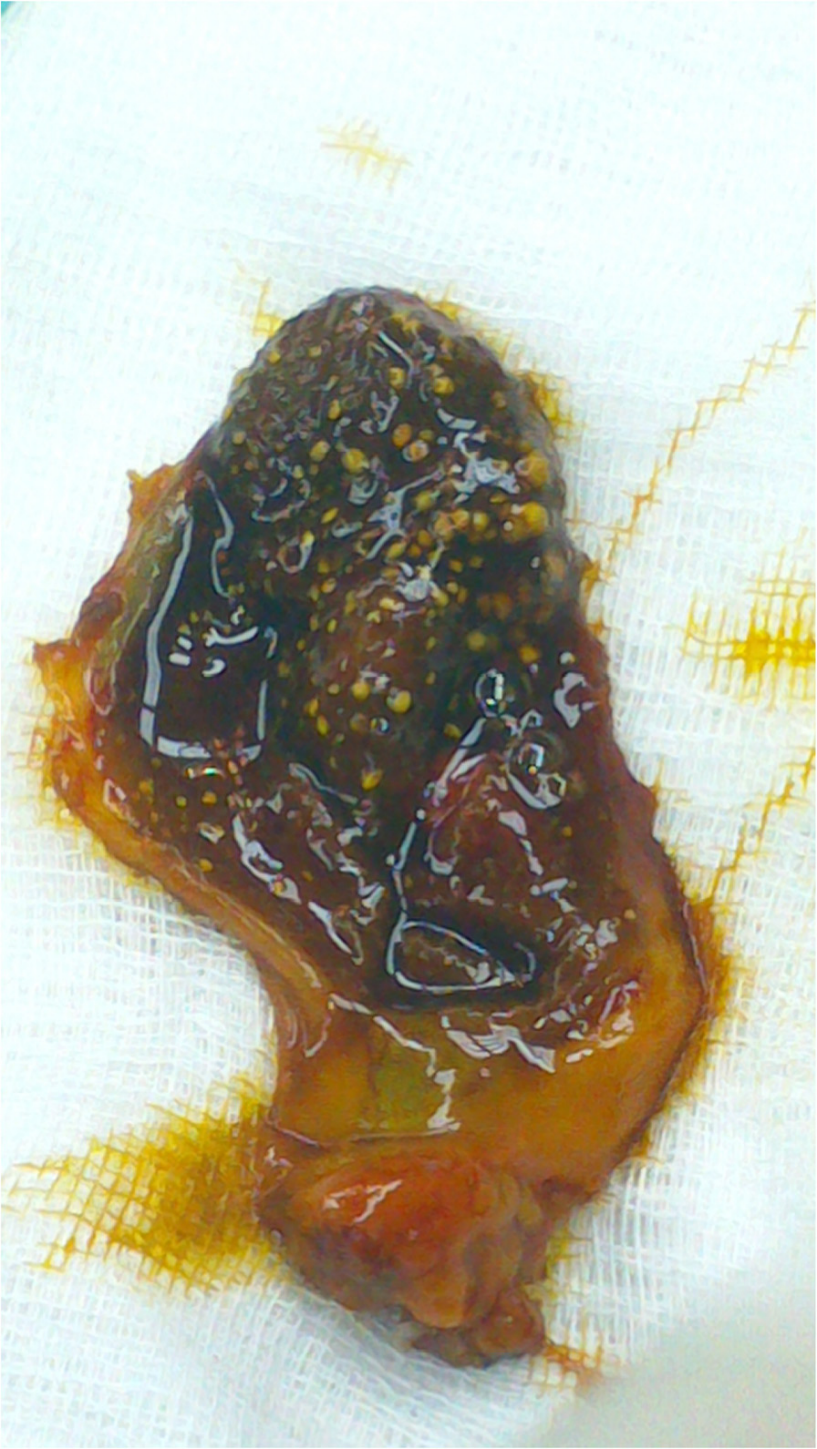
Macroscopic view of the gallbladder filled with small cholesterol gallstones after laparoscopic cholecystectomy

On macroscopic examination, her gallbladder was filled with multiple cholesterol gallstones of very small diameter, adherent to the organ's mucosa, accompanied with biliary mud (Fig. 1), which was confirmed by pathology examination.

**Fig. 2:**
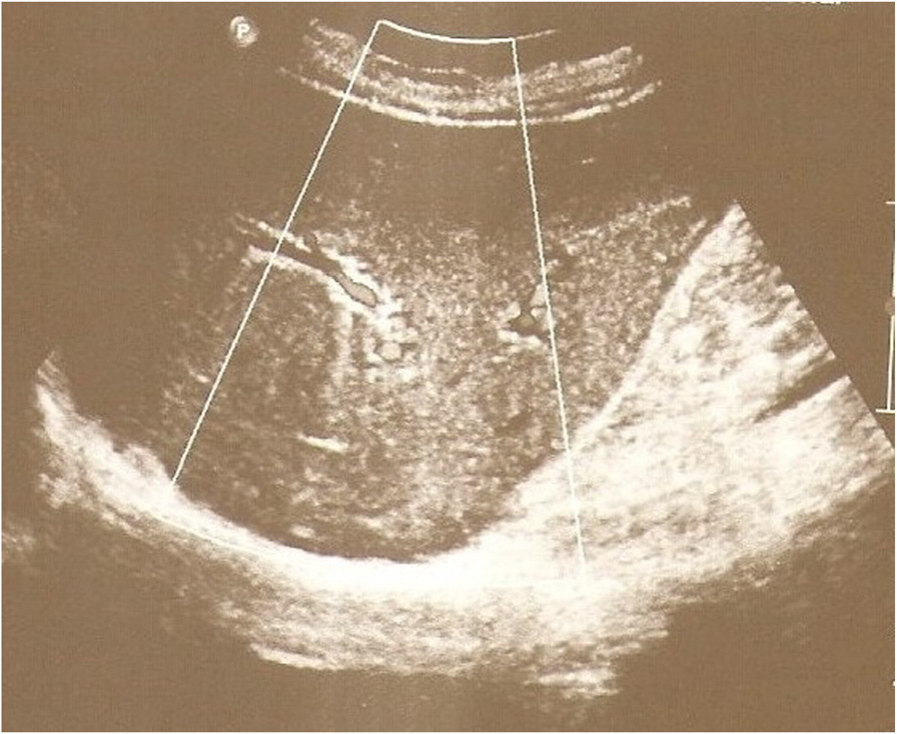
Ultrasound image shows mild dilatation of intrahepatic biliary ducts

**Fig. 3:**
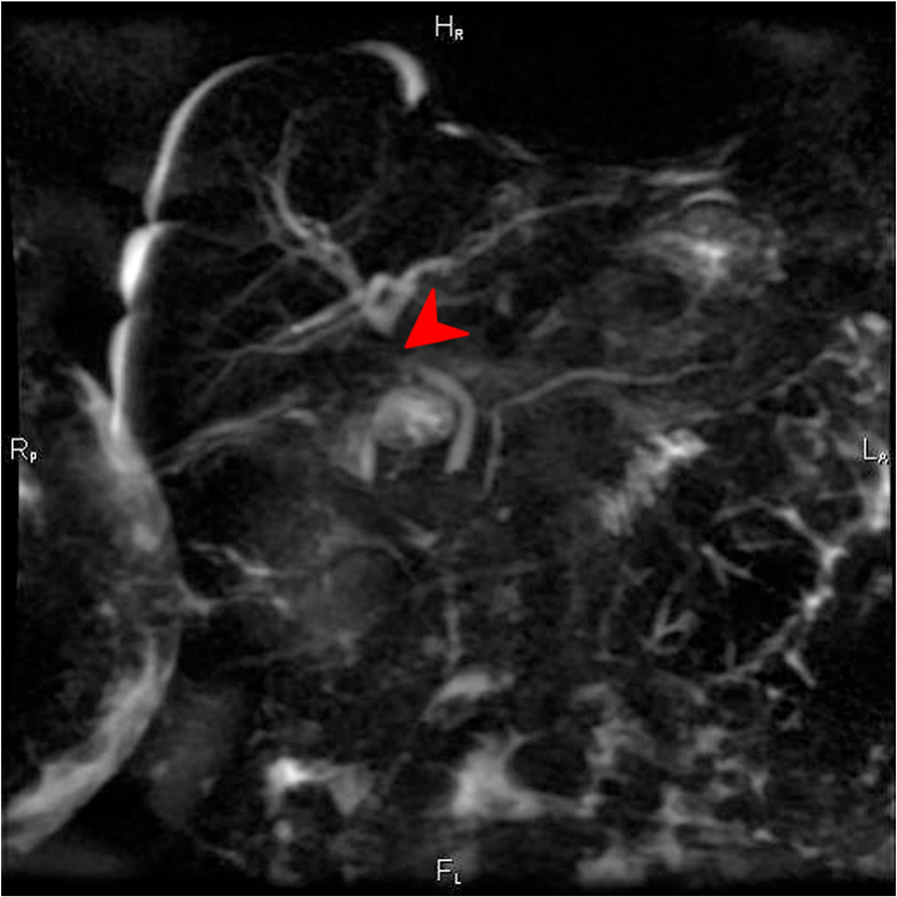
Magnetic resonance cholangiopancreatography image reveals stenosis of the common hepatic duct (*red arrow*)

On the second postoperative day, her total bilirubin level increased to 2.9 mg/dL (normal range 0.3 to 1.2 mg/dL) with conjugated bilirubin level of approximately 1.7 mg/dL (normal range <0.2 mg/dL). The following postoperative days, she gradually presented jaundice; a blood examination revealed a gradual increase in her total bilirubin level from 2.9 mg/dL to 5.2 mg/dL and an increase in her direct bilirubin level from 1.7 mg/dL to 4.4 mg/dL. On the third postoperative day, an US of her abdomen and of the biliary tree was performed which revealed a mild dilatation of intrahepatic biliary ducts (Fig. 2). Magnetic resonance cholangiopancreatography (MRCP) was then carried out showing mild dilatation of intrahepatic biliary tree close to her liver portal and a suspicion of mild stenosis of her common hepatic duct; the diameter of her common biliary duct was approximately 4 mm without dilatation, presence of gallstones, or any biloma (Fig. 3).

**Fig. 4:**
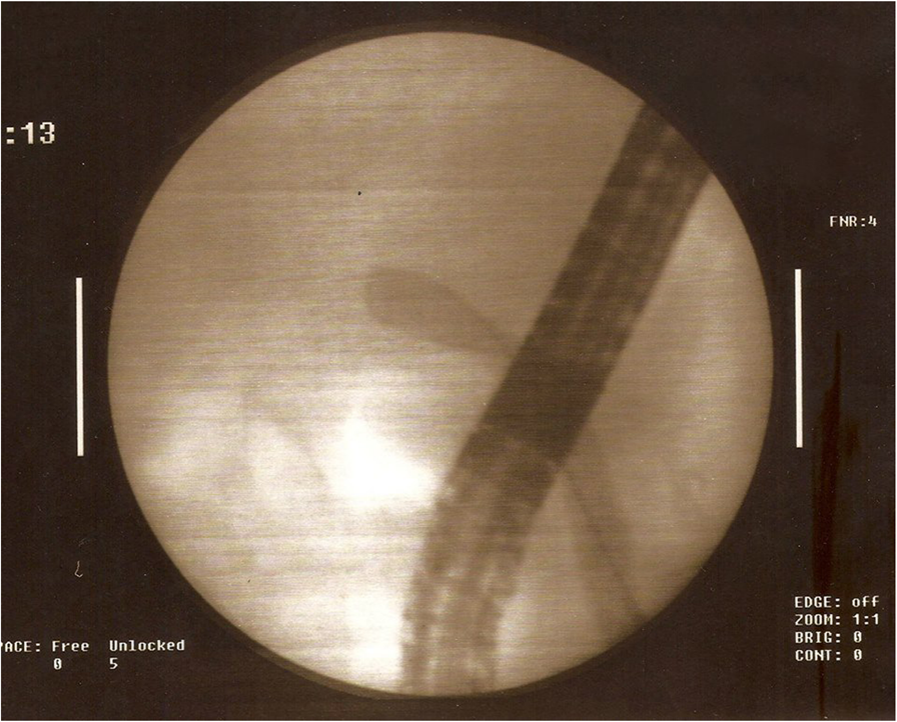
The common biliary duct presents severe stenosis to the common hepatic duct (endoscopic retrograde cholangiopancreatography image)

On the sixth postoperative day, she developed epigastric pain with localization to the right subchondral region accompanied by nausea and vomiting. A blood examination showed increased total bilirubin level of 7 mg/dL, direct bilirubin level of 4.5 mg/dL, alkaline phosphatase (ALP) of 367 U/L (normal range 30 to 120 U/L), gamma-glutamyltransferase (GGT) of 594 U/L (normal range 7 to 32 U/L), aspartate aminotransferase (AST) of 127 U/L (normal range <33 U/L), and alanine aminotransferase (ALT) of 266 U/L (normal range <31 U/L). Her clinical condition remained unchanged. After a consultation with our patient, we performed endoscopic retrograde cholangiopancreatography (ERCP) on the seventh day of her hospitalization which revealed a severe stenosis of her common biliary duct, a fact possibly attributed to polymeric laparoscopic clip (Fig. 4).

After ERCP, acute pancreatitis presented as a complication with acute pain to her upper abdomen, and she experienced nausea and vomiting. A blood test showed increased levels of total bilirubin (8.6 mg/dL), direct bilirubin (5.5 mg/dL), ALP (379 U/L), GGT (625 U/L), and her serum amylase level was 2049 U/L (normal range 28 to 110 U/L). Moreover, her urine amylase level measured approximately 19,957 U/L (normal range 42 to 321 U/L). We administered a combination of cefoxitin, ciprofloxacin, somatostatin, and analgesics for acute pancreatitis management. During the next 3 days, her serum levels of amylase gradually decreased from 953 U/L to 180 U/L until they reached a normal level of 99 U/L and her urine amylase decreased to 153 U/L. Inflammatory markers were decreased, too. Simultaneously, her total bilirubin level increased to 12.7 mg/dL with direct bilirubin level of 7.9 mg/dL. On the tenth day of hospitalization, a magnetic resonance imaging (MRI) of her abdomen was carried out which revealed inflammatory elements around her liver and to the gallbladder bed, without any image of active acute pancreatitis so she underwent an operation.

**Fig. 5:**
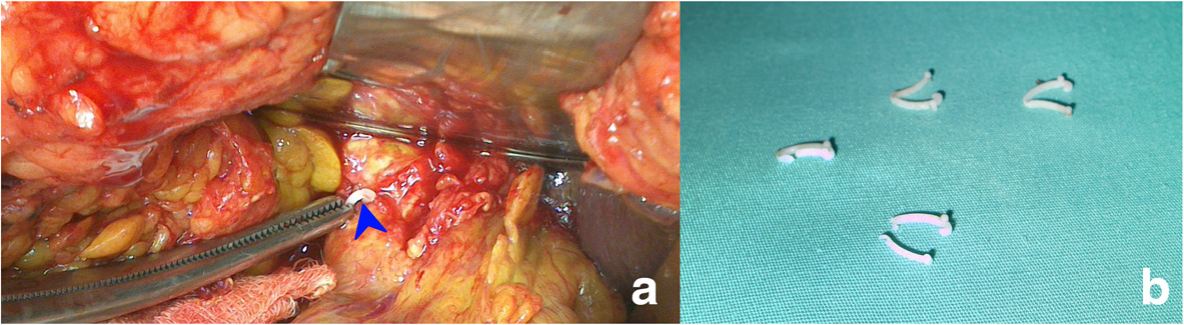
**a** Intraoperative view of the plastic laparoscopic clip close to the common hepatic duct (*blue arrow*). **b** Macroscopic view of the four removed plastic laparoscopic clips

After a preoperative consultation with our patient, our next step was to conduct an exploratory laparoscopic surgery in order to release the obstruction of her common biliary duct. We started the exploratory laparoscopy but the coexistence of the remaining inflammation of the acute pancreatitis and the local edema from the previous surgery resulted in our converting to open exploratory laparotomy (Fig. 5a). After careful dissection, we noted step-by-step all the four polymeric surgical clips, removed them, and placed drainage (Fig. 5b).

Postoperatively, she had a quick uneventful recovery. The first postoperative day, her total bilirubin level rapidly decreased to 5.7 mg/dL and her direct bilirubin level to 3.1 mg/dL and, then, they gradually decreased until they returned to within the normal range. The drainage was removed and she was discharged without presenting any further complications.

## Discussion

PCS is a medical heterogeneous condition which occurs after laparoscopic cholecystectomy and includes a variety of biliary and non-biliary disorders. The basic extrabiliary disorders are esophagitis, peptic ulcers, irritable bowel syndrome, and chronic pancreatitis. The biliary disorders include retained or dropped gallstones, biliary duct obstruction, long cystic duct remnant, biliary strictures, chronic biloma, stenosis of the sphincter of Oddi, and bile salt-induced diarrhea or gastritis. This syndrome presents an incidence of 0.4 to 4 % and the onset of symptoms ranges from 2 days to 25 years. In patients with PCS, a wide range of nonspecific symptoms may be noted, including right upper quadrant or epigastric pain occurring after meals, jaundice, diarrhea, or dyspeptic symptoms [2, 3].

The diagnostic algorithm includes US, followed by ERCP, as the gold standard. First, a US is carried out as it is capable of differentiating between non-obstructive and obstructive jaundice. Moreover, it is generally accepted that ERCP is the gold standard examination for papilla, pancreas, and biliary system visualization, particularly in patients with serum bilirubin levels of >3.0 mg/dL, with 100 % sensitivity and 95 % specificity. It reveals with accuracy the extent of stenosis or obstruction of the common hepatic duct, gallstone size, malignancies, biliobiliary fistulas, and duodenal or pancreatic pathology. It also allows therapeutic interventions such as sphincterotomy, balloon dilatation, bile drainage stone extraction, and stenting of biliary ducts avoiding iatrogenic intraoperative bile duct injury. However, ERCP is associated with significant complications such as acute pancreatitis, with an incidence of 5 % in low risk patients and 40 % in high risk patients, while severe pancreatitis with necrosis, multiorgan failure, and death is presented in less than 1 % of the patients [3, 4]. Moreover, MRCP is another noninvasive alternative method for evaluating the biliary tree in patients with PCS. MRCP can demonstrate the causes of extrinsic compression; it can reveal the presence and the level of an obstruction with 95 % sensitivity and 97 % specificity [5].

Mirizzi first described Mirizzi syndrome as a partial or total biliary obstruction to a physiological sphincter of the hepatic duct in 1948 [5]. Nowadays, it refers to common hepatic duct obstruction caused by an extrinsic mechanical compression from an impacted stone in the cystic duct or Hartmann's pouch of the gallbladder, or by inflammation in this region [6]. Post-cholecystectomy Mirizzi syndrome is a PCS disorder which can be caused by multiple mechanisms. Bile duct stones can form in a remnant cystic duct and lead to the development of this syndrome. Furthermore, a long remnant cystic duct is usually parallel to the common hepatic duct, which can easily result in Mirizzi syndrome. Mechanical obstruction of the common hepatic duct by laparoscopic clip migration or secondary inflammation is possible, too. Obstructive biliary injury can also be caused by the gradual migration of a polymeric surgical clip to the cystic duct or artery as presented in our case report.

Mirizzi syndrome does not have a specific clinical or laboratory presentation so its preoperative diagnosis is difficult. Patients with Mirizzi syndrome usually present with surgical obstructive jaundice, right upper quadrant abdominal pain, nausea and vomiting, fever, diarrhea, or recurrent cholangitis. Laboratory results can show an increase in the serum concentration of ALP and bilirubin and other liver function tests in over 90 % of patients [5, 6].

The standard treatment of Mirizzi syndrome has been open surgery in order to dissect the biliary structures, remove gallstones or relieve obstruction, identify the common duct, and ensure bile drainage. However, the type of surgical procedure is chosen depending on the type of syndrome and the degree of inflammation [5]. The laparoscopic management of Mirizzi syndrome remains a challenge for surgeons. The main reason is the dense adhesions and edematous inflammation in the Calot's triangle which causes distortion of the normal anatomy and increases the risk for biliary injury. Erben *et al*. referred to a 67 % conversion rate from laparoscopic cholecystectomy to open cholecystectomy for the treatment of Mirizzi syndrome [6].

Migration of surgical clips is a rare complication of laparoscopic cholecystectomy, which can be a cause of iatrogenic Mirizzi syndrome [7]. Studies showed that acute biliary obstruction with laparoscopic cholecystectomy is twice as common as acute biliary obstruction with open cholecystectomy because of misplaced or migrating surgical clips [8]. This clip migration takes place in the common bile duct days or years after surgery [7, 9, 10]. The exact mechanism is not known; ineffective clip placement, placement of more than four clips on the cystic duct, inflammation and necrosis around the biliary tree, increased intra-abdominal pressure, and cholecystectomy during acute cholecystitis or pancreatitis are some possible causes. Surgical clip migration can cause obstructive jaundice (37.7 %), choledocholithiasis, cholangitis with sepsis (27.5 %), biliary colic (18.8 %), acute pancreatitis (8.7 %), clip embolism, and duodenal ulcer [7, 9–11]. Goshi *et al*. proposed as a possible mechanism the compression of the clipped cystic duct by the liver; the cystic duct and clips then become inverted into the lumen of the common bile duct [11]. In our case, we consider the probable cause to be the different application mechanism of polymeric clips in comparison to the placement of metallic clips which are usually used.

We searched the literature in PubMed using the key words “post-cholecystectomy syndrome and migration of surgical clips” for the period 2000 to 2015. We found 23 papers that described multiple types of biliary PCS caused by the migration of laparoscopic clips. The causes of migration are still unclear. Not only are there multiple mechanisms of clip migration but we also have to consider ineffective technical clip application and its technical characteristics. Our case report contributes to the literature because it is the only one that refers to the migration of a polymeric plastic surgical clip in the first postoperative days; all the others referred to PCS that occurred months or years after laparoscopic cholecystectomy. Moreover, this case report is the first which clearly describes a Mirizzi type of PCS. It is also important to state that all but one of the studies on PCS referred to metallic clips (Table 1).

**Table 1:** Literature on post-cholecystectomy syndrome induced by clip migration

Authors and Reference number	Post-cholecystectomy syndrome pathology by clip migration	Onset of post-cholecystectomy syndrome	Type of laparoscopic clip
Sharma *et al*. [10]	Cholangitis associated with choledocholithiasis	2 years	Metallic
Photi *et al*. [9]	Cholangitis	9 years	Metallic
Hong *et al*. [12]	Choledochoduodenal fistula	10 years	Metallic
Ray *et al*. [13]	Stone formation in the bile duct	6 years	Metallic
Song *et al*. [14]	Cholangitis	16 years	Metallic
Baldomà España *et al*. [15]	Cholangitis	1 year	Metallic
Tseng *et al*. [16]	Bile stone with a clip in the center	10 years	Metallic
Gonzalez *et al*. [17]	Bile stone containing clip	14 years	Metallic
Goshi *et al*. [11]	Cholangitis	6 years	Not mentioned
Rajendra *et al*. [18]	Cholangitis	14 years	Metallic
Samim *et al*. [19]	Clip in duodenal ulcer bed	15 years	Metallic
Dolay *et al*. [20]	Obstructive jaundice and acute biliary pancreatitis due to choledocholithiasis	6 months	Metallic
Attwell and Hawes [21]	Biliary stricture	6 years	Endoscopic stent – surgical sutures
Steffen *et al*. [22]	Cholangitis	15 years	Metallic
Ahn *et al*. [23]	Common bile duct stones were formed around clips, and clips penetrated into the common hepatic duct	1 year	Metallic clip
Kissmeyer-Nielsen and Kiil [24]	Cholangitis	2 months	Polymeric
Mouzas *et al*. [25]	Choloperitoneum after rupture of a secondary bile duct and bile leakage	6 years	Metallic
Khanna and Vij [26]	Obstructive jaundice	5 years	Metallic
Angel *et al*. [27]	Cholangitis	7 months	Metallic
Hai *et al*. [28]	Solid mass in the common hepatic duct	6 years	Metallic
Tsumura *et al*. [29]	Bile leakage	5 years	Metallic
Yoshizumi *et al*. [30]	Choledocholithiasis	1 year	Metallic
Matsumoto *et al*. [31]	Choledochal stenosis	1 year	Metallic

## Conclusions

Laparoscopic cholecystectomy is the gold standard treatment for gallstone disease. PCS is a possible complication and it includes many types of biliary and non-biliary disorders. The migration of laparoscopic clips is one rare cause of PCS but it is also well recognized. Ineffective clip placement or clip migration can cause postoperative Mirizzi syndrome which requires early management as it can imply an acute and dramatic disorder. The management is immediate removal of surgical clips by laparoscopic approach with a high possibility of success. Laparoscopic cholecystectomy is a successful surgical approach to biliary disease but we should bear in mind the possibility of serious complications, such as Mirizzi syndrome caused by laparoscopic clip migration, as they need immediate and effective surgical management.

## Abbreviations

ALP: alkaline phosphatase
ALT: alanine aminotransferase
AST: aspartate aminotransferase
ERCP: endoscopic retrograde cholangiopancreatography
GGT: gamma-glutamyltransferase
LDH: lactate dehydrogenase
MRCP: magnetic resonance cholangiopancreatography
MRI: magnetic resonance imaging
PCS: post-cholecystectomy syndrome
US: ultrasound
